# The public’s perceptions of patient safety in healthcare

**DOI:** 10.1186/s13584-025-00691-7

**Published:** 2025-05-23

**Authors:** Ilya Kagan, Dana Arad, Joseph Mendlovic, Yossi Tal, Yaron Niv

**Affiliations:** 1 Nursing Department, Ashkelon Academic College, Ashkelon, Israel; 2 Innovation department, Clalit, Tel Aviv, Israel; 3 Nursing Department, The Academic College of Tel Aviv Yafo, Tel Aviv Yafo, Israel; 4 Ministry of Health, Jerusalem, Israel; 5 Consultant for Patient Safety, Tal Advising Services, Ramat-Hasharon, Israel; 6 Adelson Faculty of Medicine, Ariel University, Ariel, Israel

**Keywords:** Public, Views, Patient safety, Opinion, Medical errors, Patients

## Abstract

**Background:**

Patient safety during medical treatment is a central issue for health policymakers and medical teams. In this context, both the Israeli and global health systems, are witnessing an increase in the appreciation of the importance of safety indicators for quantitative measurement of treatment safety. Although an important consideration, we did not find any studies of public perception of this important topic. This study was therefore designed to examine the views and opinions of the public concerning patient safety in the Israeli healthcare system with the aim to serve as an important input in determining patient safety goals and policies.

**Methods:**

A digital questionnaire was distributed to 620 Israeli citizens, 18 years of age or older, who were randomly sampled from a pool of 75,000 citizens of Jewish origin stratified by gender, age, and area of residence.

**Results:**

Only 18.8% of the sample considered the healthcare system to be transparent in reporting and dealing with medical errors, while 23.6% reported receiving an explanation of the risks and side effects of medications before prescription. Only 56.4% reported receiving information about the risks related to surgeries and invasive operations, 62.2% claimed to understand the given explanation, and 61.5% reported going through a proper process of patient identification before a test or medical procedure.

**Conclusion:**

Patient safety is a significant concern for the public whose perceptions should be considered when planning improvements to the healthcare system. Healthcare providers must consider patients’ perceptions of patient safety issues and remain vigilant in identifying and minimizing risks associated with medical care and in verifying patient comprehension accordingly.

## Introduction

Patient safety is an essential component in healthcare. The value basis for therapeutic and medical activity, and hence for creating a culture of patient safety, is the Hippocratic injunction of *Primum non-Nocere* (First do no harm).^1^ Efforts to improve patient safety in healthcare may include quality and patient safety training, root cause analysis following errors, and team huddles.^2^.The promotion of patient safety requires patient engagement and involvement in care management and decision-making at all organizational levels; from identifying errors and failures, learning and drawing lessons, and initiating processes to improve and prevent errors, up to the level of policy-making. Patient awareness to the importance of safety and the need for vigilance when negotiating the health care system as health consumers are significant components in increasing patient engagement.^3^.

Transparency about medical failures and errors with patients and the public is an essential element in creating trust between the healthcare system and health consumers. Relationships of trust promote mutual respect, effective clinical intervention, and patients’ adherence to recommendations. However, there is little information or declared policy regarding patients’ awareness and involvement in safety or prevention of errors, and studies of the awareness and attitudes of the public and patients regarding safety of care are scarce. Madden et al. (2021) examined the perceptions of 584 patients regarding the safety climate in their primary care clinic.^4^ Their findings revealed generally positive feelings about the safety of their treatment, staff efficiency, and teamwork in the clinic, and a high level of patient knowledge and accountability. But this study examined patients’ attitudes regarding their treatment and not attitudes of the general public. Another study, with a focus on patients’ perceptions of safety in primary care, revealed two main factors that contribute to safe care, namely positive communication with the physician and trust in the caregiver regarding the quality of care.^5^ One report described trust as a contributing factor to promoting patient safety, while a lack of confidentiality and continuity of care were described as deleterious.^6^ Villar et al. (2020) who reviewed the 2008–2019 literature addressing patients’ perceptions of contributing factors to adverse events in hospitals concluded that poor interpersonal caregiver-patient communication, lack of continuity of care, inadequate skill training among health care providers, workload, and a lack of control and accountability, affect patient safety.^7^ Their suggestions to prevent errors included staff training, using checklists, encouraging patient participation, and accommodating the hospital environment to the patients. Lee et al. (2021)^3^ who evaluated the public perception of patient engagement in patient safety among 600 citizens in South Korea, reported a low awareness of the issues of patient safety and quality of care. A recent online study from Saudi Arabia^8^ investigated public perceptions of medical errors, highlighting misconceptions and their impact. About 31.5% of responders reported personal or indirect experiences with medical errors, with misdiagnosis cited as the most common error (55.6%). A significant negative correlation was found between the ability to differentiate errors and perceptions of their prevalence. The study findings emphasized the public’s confusion regarding medical errors and complications.

Our study was designed to fill the gap left by the lack of published studies addressing the general public’s perceptions and views about patient safety issues, which we consider should be included in plans to improve patient safety. We believe that investigating and understanding the attitudes of the general public regarding safety will promote trust in healthcare and increase the involvement of patients in decision-making and care management.

### Aim

This study aimed to examine the views and opinions of the general public towards patient safety aspects of the Israeli healthcare system.

## Methods

### Sample and study design

An accredited survey firm conducted the data collection process. A digital questionnaire was distributed to 620 Israeli citizens, who were randomly sampled (simple randomization) from a pool of 75,000 citizens of Jewish origin stratified by gender, age, and area of residence. The inclusion criteria were being adult (age > 18), Jewish, and Hebrew speaking, with the ability to respond to an online questionnaire, and having received medical treatment during the 12 months preceding the survey. The exclusion criteria were volunteers who could not respond to an online questionnaire such as people without a smartphone. A total of 500 (80.64%) questionnaires were completed. The data collected were anonymized with no identification of patient names, medical institutions, organizations, or specific therapists. The purpose of the study was presented to each participant.

### Tools

In the absence of any validated questionnaires, we decided to develop a dedicated questionnaire that could be used to examine the general public perceptions of patient safety. A panel of five experts in Patient Safety and Quality in Care reviewed and validated the tool. Only items in full consensus were included. The 41 initial items were reduced to 33 during the validation process (see Table 1). These examine the perceptions of: (1) incidence of medical errors, (2) patient education and explanations about risks before a medical procedure, (3) patient identification, (4) knowledge about the Patient’s Rights Act and quality indicators, (5) management of medical errors in healthcare, (6) importance of disclosing information about medical errors, (7) the main causes of medical errors, (8) activities to reduce errors, and (9) socio-demographic characteristics.

**Table 1:** Background characteristics of the participants (*n* = 500)

Variables	Category	*N*	Valid %
**Gender**	Male	241	48.2
	Female	259	51.8
**Family status**	Single	126	25.2
	Married	314	62.8
	Divorced	49	9.8
	Widowed	11	2.2
**Religiosity**	Secular	283	56.6
	Traditional	109	21.8
	Religious	66	13.2
	Ultra-orthodox	42	8.4
**Income level** (Compared to average family income)	Way below the average	108	21.6
	Below the average	104	20.8
	The average	128	25.6
	Above the average	77	15.4
	Way above the average	38	7.6
	Not interested in answering	41	8.2
	Missing	4	0.8
**Perceived health**	Excellent	123	24.6
	Very good	262	52.4
	Good	90	18.0
	Fair	22	4.4
	Poor	3	0.6
**Place of birth**	Israel	362	76.7
	East Europe	14	3.0
	Middle East & Asia	12	3.3
	North & South America	14	2.9
	Former Soviet Union	54	11.8
	Western Europe	12	2.5
**Education**	Elementary school	52	10.4
	High school education	116	23.2
	Academic education	228	46.8

*The incidence of medical errors* as reported by participants was measured by seven items. The participants were asked to use a scale of 1 (totally disagree) to 5 (totally agree) to rate statements referring to the frequency of medical errors: during hospitalization; in community and primary care; in medical diagnosis; in medication; during invasive procedures and surgeries; and in routine care by caregivers and by patients. The final score was calculated as the mean, where a higher score reflects a higher incidence of errors (lower patient safety). The internal reliability score (Cronbach’s alpha) for this part was 0.85.

*Patient education and explanations about possible risks* before receiving medical treatment during the past year were measured using four items. The participants were asked to report the degree to which they received information and explanations about risks before treatment according to a scale from 1 (not at all) to 5 (to a very large extent). The final score was calculated as the mean, where a higher score reflects more information and explanations received. The Cronbach’s alpha for this part was 0.76.

The application of the procedures for *patient identification* by caregivers was tested by one question, rated on a scale from 1 (not at all) to 5 (to a very large extent): “At your last healthcare visit, did a physician or a nurse ask for your name and ID number?” A higher score reflects better practice of correct patient identification.

*Knowledge about the Patient’s Rights Act and quality indicators* within the National Quality Indicators Program was measured using two questions: “To what extent are you familiar with the Patient’s Rights Act?” and “Are you familiar with the quality indicators that the Ministry of Health publishes for hospitals?” The responses to the questions were measured using a scale ranging between 1 (not at all) and 5 (definitely).

*Dealing with medical errors and transparency of the healthcare organization* was measured using two items. The first statement referred to the degree of effort invested in the prevention of medical errors by the health system: “The health system does what is necessary to prevent medical errors”. The second item required the participants to consider the degree of transparency of the healthcare system regarding medical errors: “The health system in Israel practices transparency in reporting and dealing with medical errors”. The participants were asked to rate these statements on a scale from 1 (totally disagree) to 5 (totally agree).

*The main causes of medical errors* in the healthcare system were measured using seven items representing possible causes for the development of adverse events during medical care. The participants were asked to rate these statements on a scale from 1 (totally disagree) to 5 (totally agree). The Cronbach’s alpha for this section of the tool was 0.76.

*Activities to reduce errors* were assessed using nine statements representing actions known to reduce the rate of failures in medical care. The participants were asked to rate these statements on a scale from 1 (totally disagree) to 5 (totally agree). Cronbach’s alpha for this section was 0.82.

*The importance of information regarding medical errors* from the participant’s point of view was measured using the following statement: “I need to know the rate of errors and failures in the medical institution in which I am treated”. The participants were asked to rank the response on a scale from 1 (not at all) to 5 (to a very large extent).

*Personal and socio-demographic characteristics* included gender, age, religion, marital status, country of birth, and education. One question required participants to rate their health according to a scale between 1 (bad) and 5 (excellent). The previous year’s exposure to medical services was measured by a question: “In the last 12 months, have you received medical treatment (from a doctor, nurse, other therapists, in a hospital, or in the community)?”

### Data collection

The research and data collection were conducted in collaboration with The Dialog - Organizational Consulting Research and Training LTD (ISO/IEC 27001:2013, registration number 109502). The firm conducts surveys for a variety of clients in the public and private sectors. Surveyors with appropriate qualifications and extensive experience in conducting surveys received a comprehensive briefing as well as clear instructions before data collection, and their performance was strictly supervised by the researcher responsible for the study. Participants in the database have responded previously to these surveys with great success. Assuming that online survey responses are sufficient, a survey is a fast and effective tool that is convenient for participants and surveyors, ensures confidentiality, and allows for a broad population representation.

### Ethical considerations

The data were collected through the online polling panel. In Israel, public opinion surveys do not require ethical approval. However, at the beginning of the questionnaire, participants were given an explanation about the survey. Answering the questionnaire was considered consent to participate. The researchers received a data file with no personal details of the participants.

### Data analysis

Descriptive statistics were used to test the distribution of socio-demographic and study variables. The normality of the distribution of the dependent variables was tested as follows. The Kolmogorov-Smirnov test of normality failed to support the normality assumption, which is very common in such cases. However, descriptive statistics for the outcome variables indicated mild skewness and Kurtosis for Perceived Dealing: Skewness = -0.088; Kurtosis = -0.704, and Perceived Transparency: Skewness = 0.247; Kurtosis = -0.782 suggested that a normality assumption was reasonable. Moreover, given our relatively large sample size (*n* = 500), we assessed the normality of variable distributions primarily through visual inspection of histograms and Q-Q plots. Based on our visual inspections, we concluded that the distributions of our variables were sufficiently close to normal to proceed with the chosen parametric statistical analyses. Pearson correlations were used as a preliminary test of associations between variables. Multivariate regression models were developed to explain respondents’ perceptions of the healthcare system’s commitment to preventing medical errors (Model 1) and ensure transparency in reporting medical errors (Model 2) using respondents’ background characteristics, knowledge, and engagement with the public system. These models were mainly explorative, as no previous studies on the matter have been published. The data were analyzed using SPSS v.28 (IBM, US).

## Results

### Participants

All the participants were Jewish, and 56.6% (283) reported being secular. The average age was 46.88 years (SD = 17.98, range 18–88), 48.2% (241) of the sample were men, and 62.8% (314) were married. Of the sample, 40.2% had an academic education, 42.4% earned below the average wage in the country, 76.7% (362) were Israeli born; with 0–11 children in the family (M = 1.52, SD = 1.85, Mode = 2). Other socio-demographic and professional characteristics are presented in Table 2.

**Table 2:** The distribution of the participants’ responses (*n* = 500)

**Sections and items**	**M**	**SD**	**Scale (valid **%**)**				
			**1**	**2**	**3**	**4**	**5**
**Incidence of medical errors** - from 1 (not at all) to 5 (to a very large extent)							
1. Medical errors in hospitals are common	3.14	0.94	17 (3.4)	96 (19.2)	232 (46.4)	111 (22.2)	44 (8.8)
2. Medical errors in community health services are common	3.12	0.91	11 (2.2)	113 (22.6)	219 (43.8)	121 (24.2)	36 (7.2)
3. Errors in a medical diagnosis are common	3.20	0.93	11 (2.2)	101 (20.2)	206 (41.2)	140 (28.0)	42 (7.2)
4. Medication errors are common	2.78	1.00	45 (9.0)	154 (30.8)	192 (38.4)	83 (16.6)	26 (5.2)
5. Medical errors in performing surgeries are common	2.64	1.01	51 (10.2)	197 (39.4)	161 (32.2)	63 (12.6)	28 (5.6)
6. Caregivers (doctors, nurses and other health professionals) make errors	2.97	0.94	20 (4.0)	133 (26.6)	220 (44.0)	94 (18.8)	33 (6.6)
7. Patients make and contribute to errors and failures in a medical care	2.76	1.03	58 (11.6)	143 (28.6)	184 (36.8)	93 (18.6)	22 (4.4)
**Patient education and explanations about risks** - from 1 (not at all) to 5 (to a very large extent)							
8. When you received a medication, to what extent was it explained to you why you were receiving it?	3.45	1.22	44 (9.1)	68 (14.0)	101 (20.8)	170 (35.0)	103 (21.2)
9. To what extent did you receive an explanation of the side effects and risks, when you last received a prescription for a medication?	2.26	1.37	212 (43.7)	89 (18.4)	70 (14.4)	73 (15.1)	41 (8.5)
10. When you had surgery or another invasive procedure, to what extent were the possible risks explained to you?	3.58	1.21	26 (7.5)	42 (12.2)	72 (20.9)	117 (33.9)	88 (25.5)
11. To what extent did you understand the explanation given to you before the procedure?	3.69	1.10	15 (3.9)	45 (11.6)	87 (22.4)	141 (36.2)	101 (26.0)
**Patient identification** - from 1 (not at all) to 5 (to a very large extent)							
12. At your last visit for a medical examination, did a physician or nurse ask for your name and ID number?	3.55	1.56	94 (19.6)	44 (9.2)	47 (9.8)	95 (19.8)	200 (41.7)
**Knowledge about Patient’s Rights Act and quality indicators** - from 1 (not at all) to 5 (to a very large extent)							
13. To what extent do you know about the Patient’s Rights Act?	2.32	1.22	164 (33.5)	127 (25.9)	104 (21.2)	68 (13.9)	27 (5.5)
14. Are you familiar with the quality indicators published by the Ministry of Health for the hospitals? Yes or No.	1.81	0.39	Yes 93 (18.6)	No 407 (81.4)			
**Sections and items**	**M**	**SD**	**Frequency (valid %)**				
			**1**	**2**	**3**	**4**	**5**
**The main causes of medical errors** - from 1 (totally disagree) to 5 (totally agree)							
15. Lack of resources	4.04	1.12	23 (4.8)	24 (5.1)	78 (16.4)	137 (28.8)	213 (44.8)
16. Lack of knowledge among caregivers	2.92	1.14	51 (10.8)	131 (27.7)	142 (30.0)	105 (22.2)	44 (9.3)
17. The Israeli “it will be fine” culture	3.53	1.16	32 (6.7)	54 (11.3)	129 (27.0)	152 (31.9)	110 (23.1)
18. Carelessness of the caregivers	2.68	1.22	90 (18.7)	139 (28.8)	137 (28.4)	66 (13.7)	50 (10.4)
19. Burnout and fatigue among caregivers	4.34	0.93	6 (1.2)	21 (4.4)	53 (11.0)	124 (25.8)	277 (57.6)
20. The diseases are serious and the patients’ condition is complex	3.21	1.14	40 (8.5)	83 (17.6)	154 (32.7)	128 (27.2)	66 (14.0)
21. Inadequate learning from errors	3.68	1.04	13 (2.9)	37 (8.2)	151 (33.3)	133 (29.4)	119 (26.3)
**Activities to reduce medical errors** - from 1 (totally disagree) to 5 (totally agree)							
22. Increasing professional personnel	4.66	0.73	3 (0.6)	7 (1.4)	30 (6.0)	88 (17.6)	363 (72.6)
23. Adopting an organizational culture of taking responsibility	4.55	0.82	1 (0.2)	13 (2.6)	42 (8.4)	113 (22.6)	314 (62.8)
24. Patient engagement and involvement in medical decision-making	4.26	1.02	10 (2.0)	22 (4.4)	73 (14.6)	134 (26.8)	246 (49.2)
25. Punishment of caregivers involved in medical errors	3.84	1.30	26 (5.2)	62 (12.4)	98 (19.6)	119 (23.8)	168 (33.6)
26. Drawing lessons from mistakes to prevent the recurrence of errors	4.71	0.70	2 (0.4)	4 (0.8)	29 (5.8)	85 (17.0)	362 (72.4)
27. Advancing technologies to improve patient safety	4.68	0.71	2 (0.4)	8 (1.6)	22 (4.4)	96 (19.2)	360 (72.0)
28. Increasing the budget for error prevention and treatment	4.63	0.75	3 (0.6)	2 (0.4)	41 (8.2)	103 (20.6)	334 (66.8)
29. Sharing knowledge between organizations regarding errors	4.70	0.66	-	3 (0.6)	35 (7.0)	82 (16.4)	369 (73.8)
30. Full transparency regarding medical errors	4.69	0.69	1 (0.2)	4 (0.8)	30 (6.0)	97 (19.4)	350 (70.0)
**Importance of information regarding medical errors** - from 1 (totally disagree) to 5 (totally agree)							
31. It is important for me to know the rate of errors and failures in the medical institution in which I am treated	4.46	0.90	2 (0.4)	18 (4.0)	58 (11.6)	110 (22.0)	295 (59.0)
**Dealing with errors and failures** - from 1 (totally disagree) to 5 (totally agree)							
32. The health system does what is necessary to prevent errors and failures	3.02	1.08	44 (8.8)	117 (23.4)	162 (32.4)	139 (27.8)	38 (7.6)
33. The health system practices transparency regarding dealing with errors	2.49	1.11	114 (22.8)	140 (28.0)	152 (30.4)	77 (15.4)	17 (3.4)

### Descriptive statistics

Almost all (95%) of participants have received medical treatment of some sort during the last 12 months. This result was similar for men and women (95% and 96%, respectively) and all age groups (93% and 96% in the age groups 18–64 and 65+, respectively). Similarly, there were no statistically significant differences in medical treatment frequency between geographical districts (range of 92–100%). A total of 77% defined their physical status as excellent or good (men and women alike). As expected, this number decreased with age, with no statistically significant differences between the center and periphery of the country.

The responses to the questions and items and the analysis are presented in Table 1. Regarding processes to ensure safe care in the field, from the patient’s point of view, there are a number of issues requiring significant improvement. Only 18.8% of the sample considered that the system practices transparency regarding reporting and dealing with errors, while only 23.6% reported receiving an explanation of risks and side effects of prescribed medications. In contrast, 56.4% of the participants reported receiving information regarding risks related to surgeries and invasive operations although only 62.2% claimed to understand the given explanation. Only 61.5% of the study population reported going through a process of patient identification prior to a test or medical procedure.

The findings are similarly discouraging regarding knowledge of quality improvement and measurement processes at the national level. Only 19.4% of the participants said they were familiar with the Patient’s Rights Law, and 81.4% were not familiar with the quality indicators published by the Ministry of Health (MOH). Ninety-three participants who were familiar with the MOH quality indicators program, checked the ranking position of the hospital, in which they underwent the invasive procedure, in the ranking list of quality indicators published by the Ministry of Health. Therefore, only 4.5% of the study sample claimed that they verified the quality ranking of the treating hospital.

There were interesting findings regarding the factors affecting the occurrence of errors and the processes for reducing adverse events. The public demonstrated a high level of awareness of the difficulties and challenges of the health system regarding the shortage of resources, lack of personnel, poor organizational culture, burnout and fatigue among the staff, and insufficient efforts invested in learning from failures and errors. However, the results indicated that the public places a high value to the level of knowledge and care of the staff at the patient’s bedside. Similarly, the suggestions for reducing errors in the health system emphasized the importance of increasing the budget and adding personnel, strengthening the culture of taking responsibility, improving organizational learning processes, and increasing transparency and patient involvement in making medical decisions. Despite the appreciation of their difficulties, the results revealed a judgmental and negative attitude and the expectation that professionals, responsible for medical errors and failures should be punished.

### Relationships between the study variables

As shown in Table 3, there were gender differences in perceptions regarding the incidence of errors, patient education, and activities to reduce medical errors. Interestingly, women estimated the rate of errors to be higher, reported that they received fewer explanations about risks in medical treatment, and perceived the activities to reduce errors as more effective.

**Table 3:** Gender differences in study variables (*n* = 500)

Variables	Gender	*N*	M	SD	t
Incidence of medical errors	male	241	2.85	0.67	2.93**
	female	259	3.03	0.72	
Patient education and explanations	male	232	3.27	0.99	2.66**
	female	258	3.03	0.99	
Patient identification	male	229	3.63	1.49	1.09
	female	251	3.47	1.63	
Knowledge about Patient’s Rights Act	male	236	2.22	1.25	1.75
	female	254	2.41	1.20	
Knowledge about quality indicators	male	241	1.82	0.39	0.19
	female	259	1.81	0.39	
Dealing with medical errors	male	241	3.03	1.04	0.26
	female	259	3.01	1.12	
Transparency of the system	male	241	2.52	1.14	0.64
	female	259	2.46	1.07	
Causes of medical errors	male	235	3.44	0.65	1.66
	female	254	3.54	0.66	
Activities to reduce errors	male	241	4.46	0.58	2.40*
	female	259	4.58	0.52	
Importance of information regarding medical errors	male	241	4.44	0.95	0.53
	female	259	4.48	0.86	

Notes: ** *p* <.01, * *p* <.05

Comparing analysis by country of birth revealed that Israeli-born participants perceived fewer errors (M = 2.90, SD = 0.71 versus M = 3.07, SD = 0.68, t = 2.22, *p* =.03, respectively) and judged the reasons as less significant (M = 3.43, SD = 0.64 versus M = 3.64, SD = 0.67, t = 2.94, *p* =.003, respectively) than foreign-born responders. No other statistically significant differences were found between the study variables and demographic characteristics. Correlation tests detected no statistically significant relationships between age and the study variables.

A logistic regression analysis (Table 4) was implemented to examine the degree of public trust in the commitment of the healthcare system to prevent medical errors (Model 1) and ensure transparency in reporting medical errors (Model 2). These regression models were run in steps, i.e., hierarchically; that is, new explanatory variables were entered in each step to assess their contribution to the percentage of explained outcome variability. The first step tested the possible effects of respondents’ background characteristics and revealed that gender and age did not significantly affect the outcome variables in either model. In step two, we entered the knowledge about the Patients’ Rights Act (PRA) and the public-reported incidence of medical errors index. The results indicate that these two independent variables affect both outcomes. Higher knowledge of PRA was positively associated with higher levels of the dependent variables (β = 0.09, *p* =.055; β = 0.14, *p* =.003; respectively), whereas the public-reported incidence of medical errors index was found to be negatively associated with these two outcomes (β = − 0.18, *p* <.001; β = − 0.18, *p* <.001). That is, familiarity with the Patient Rights Act increased the perception of the health system’s commitment to preventing errors and transparency in reporting. In contrast, exposure to failures and errors in their care, lowered the perception of the healthcare systems’ commitment and transparency. In a third step, the perceived commitment of the system to preventing medical errors variable was included as an additional explanatory variable of the perceived system’s transparency in reporting medical errors. The added variable was found to have a significant effect on the perceived transparency. In other words, both outcomes showed a correlation with each other. An attempt to test the interaction effect between perceived treatment and knowledge of PRA and the perceived errors index did not identify additional associations.

**Table 4:** Hierarchical regression - healthcare system’s commitment to preventing medical errors (Model 1) and demonstrating transparency in reporting medical errors (Model 2)

	Model 1			Model 2		
	b (S.E) *p*-Value	β	95%CI	b (S.E) *p*-Value	β	95%CI
Step 1						
Gender	-0.004 (0.10) 0.985	− 0.00	-0.20, 0.20	-0.04 (0.10) 0.719	− 0.02	-0.22, 0.17
Age	-0.003 (0.01) 0.282	− 0.05	-0.01, 0.003	-0.004 (0.01) 0.113	− 0.05	-0.01, 0.001
*R* ^*2*^	0.002			0.001		
Step 2						
Knowledge of the Patient Rights Act	0.08 (0.04) 0.055	0.09	-0.01, 0.003	0.12 (0.04) 0.003	0.14	0.04, 0.21
Incidence of medical errors	-0.30 (0.07) < 0.001	− 0.18	-0.49, -0.10	-0.30 (0.08) < 0.001	− 0.18	-0.48, -0.13
*ΔR* ^*2*^	0.044			0.055		
*R* ^*2*^	0.047			0.060		
Step 3						
Incidence of medical errors				0.58 (0.04) < 0.001	0.56	0.49, 0.66
*ΔR* ^*2*^				0.303		
*R* ^*2*^				0.363		

Notes:

• Model 1: Outcome is “Commitment to prevent medical errors”; Model 2: Outcome is perceived “Transparency in reporting medical errors”; *N* = 485; 95%CI is 95% confidence interval based on bias corrected bootstrapping, *n* = 1,000

• The items measuring the dependent variables:

o Commitment to prevent medical errors - “The healthcare system is doing what is necessary to prevent medical errors”

o Transparency in reporting medical errors – “The Israeli healthcare system is transparent in reporting medical errors”

## Discussion

Over the last decade, patient safety and risk management have become a major public concern that has motivated multiple initiatives to ensure that patients receive the best possible treatment without causing harm. This study was designed to assess public knowledge and his understanding of patient safety and risk management in medicine. Given the lack or paucity of information on the subject, the results of this study contribute significantly to the current body of knowledge on this important topic and identify public-perceived gaps in patient safety that may assist policymakers. Participants reported low transparency, with only 18.8% of respondents expressing a belief in the transparency of the healthcare system in handling medical errors. This was evidenced as limited risk communication, where a significant portion of respondents reported not receiving adequate information about risks associated with medications (23.6%), surgeries (56.4%), or invasive procedures (61.5%). Even when information was provided, comprehension was limited (62.2% claimed to understand). Additional issues were: inadequate patient identification, with only 61.5% of respondents reporting a proper patient identification process; a low awareness of patient rights, with a concerningly low percentage (19.4%) familiar with the Patient’s Rights Act, and even fewer aware of the quality indicators published by the Ministry of Health; and gender and birth country differences, with women more aware of errors, explanation about risks, and activities to reduce errors than men. Israeli-born participants perceived fewer errors than foreign-born participants.

Our findings revealed major gaps between the information available to the public and what is known about various aspects of patient safety, and a perceived lack or partial implementation of critical principles of patient safety by clinical caregivers. For example, the study results emphasize the need for improvement in transparency and patient comprehension of the explanations provided before prescribing medication or a surgical procedure. Interestingly women were more aware than men of the need for a better explanation of potential side effects, and the possibilities of adverse events. Previous studies have reported that health literacy is affected by multiple factors,^9^ but, gender differences may be related to consultation rates.^10^.

Significantly, a few of the participants felt that they had received an adequate explanation regarding medication side effects and risks. In contrast, more participants reported being given an explanation before surgery, although many failed to understand it. One possibility for these findings is that the information provided is insufficiently clear and depends on multiple factors including a belief in the physician’s expertise.^11^ Another option relates to the legal requirement for signed consent before a surgical procedure as opposed to the ethical requirement to provide information to a patient before providing medication. Although surgical informed consent is mandatory and includes patient-clinician discussion it does not guarantee patient comprehension.^12^ Notably, while not mandatory, patient education before prescribing medication has been related to improvements in compliance^13^ and reduction in errors.^14^ The study findings regarding low public awareness of the Patient’s Rights Act, quality measure programs, and quality improvement processes at the national level are consistent with the results of a study from South Korea that reported low public awareness of accreditation and regulatory processes for the patient safety and quality.^3^ It should be noted that this study’s objectives differed from ours, and there might be cultural biases in the findings related to some patient safety issues including the practice of identifying patients by two identifiers. The need to increase public awareness of potential failures in care and their prevention can be met by public education. At the policy-making level, it is necessary to communicate these processes to the public and to integrate information into national projects or campaigns that deal with quality measurement and improvement. These steps should focus on increasing the awareness of patient safety and encouraging patient engagement with a more active role in healthcare decisions.

The public is aware of leading causes of medical failures and errors, such as lack of resources, an organizational culture of shortcuts and non-compliance with procedures, poor learning from adverse events, along with workload and burnout among caregivers. Such systemic difficulties are widely discussed by the bodies leading the healthcare system in Israel, and are disseminated to the public by the media, and the reality of in the “field” encounters with service providers at the patient’s bedside. These practices should be encouraged and the main causes of medical errors and activities to reduce medical errors should be further studied. Comparing our results to similar surveys from Israel and other countries, could refine our understanding of the unique challenges and opportunities within the healthcare system and allow for more targeted and effective policy interventions.

In recent years, media coverage and increased transparency in healthcare have raised public awareness of safety issues, with patients and their families encouraged to question medical professionals about treatment options, procedures, and potential risks.^15^ Significantly, patient-centered questionnaires such as PREMS (Patient Reported Experience Measures) and PROMS (Patient Reported Outcome Measures) are becoming a part of evaluating the quality of care.^16^ The internet and social networks have also empowered patients to research their medical conditions and treatments. Patients can access online resources, which provide information on medical errors and how to avoid them.^17^ As a result, the public has become more aware of the right to safe care, and of the importance of reporting medical errors and adverse events. Patients and their families are becoming more involved in healthcare decision-making processes and better results and safety may be achieved by including them in discussions and decisions regarding their care. The public is also becoming more aware of the importance of reporting medical errors and adverse events and expects healthcare providers to adhere to high standards of care to minimize risks associated with medical care and provide safe and effective treatment. In recent years, there has been an increasing emphasis on patient safety in medical education in many countries, and medical schools now include patient safety and risk management courses in their curricula.^18^.

Logistic regression analysis revealed interesting findings that could reflect the shaping of public trust in the healthcare system, namely a positive relationship between knowledge of the Patient’s Rights Law and the public’s belief in the health system’s commitment to patient safety and transparency in reporting failures, and a negative relationship between these variables and exposure to failures in care. These findings suggest that public education about the Patient’s Rights Law may increase patients’ involvement in their treatment, improve trust and communication between patients and medical staff, and improve medical outcomes.

There is extensive discourse and policy regarding the transparency of surveys and data in the healthcare system’s quality improvement field. Thus, quality surveys conducted as part of the National Quality Program are published on the websites of the Ministry of Health and are even partially accessible to the public through written or online media (press). However, the issue of medical failures and errors is considered sensitive and does not receive the same degree of transparency and accessibility. Specifically, data regarding failures and medical errors are not transparent. We consider that this issue requires further consideration and would recommend increasing accessibility in order to ensure transparency and to increase awareness of the risks to patient safety in the healthcare system.

Similarly, we would recommend the Ministry of Health to lead and establish a policy regarding the transparency of data regarding the publication of information on medical errors and failures. This would include publishing patient recommendations designed to ensure safe care, including providing complete information when meeting with the health system, being accompanied by a relative or significant other when making important medical decisions, complying with the instructions of the caregivers, and transferring information from sources that are not accessible to the caregivers when receiving care.

We can conclude that policymakers and healthcare system leaders should be more precise and determined in their instructions, guidelines, and rules about being transparent in reporting and dealing with medical errors. This involves providing an explanation of the risks and side effects of medications before prescription, as well as detailed information about possible complications of operations and invasive procedures and exercising extreme caution in the process of patient identification.

### Limitations

The limitations of our study are that the study population included only Jewish participants; thus, the views of the Arab and other minority populations were not examined. Specifically, further studies are needed to assess public views on patient safety in the Arab sector. Examining the issue among Arabic speakers is essential as there may be cultural influences on the perception of medical errors and failures, the steps to deal with them, and the transparency of this issue. Future studies, should include a variable focusing on a cultural adaptation of healthcare that can affect patient safety and quality of care for minorities in Israel. Another point is that, the average age of our study population (46.88 years) is lower than that of most healthcare users, although 95% of the sample had received medical treatment in the last 12 months. The patient safety issues examined in our study were chosen by patient safety experts and may not include everything of interest to the public. A further study should investigate public perceptions regarding the factors affecting patient safety and their suggestions to improve patient safety.

### Policy implications and recommendations

The results of this study highlight critical gaps in public knowledge and awareness regarding issues concerning patient safety and the healthcare system’s activities regarding patient safety in Israel. Based on these findings and scientific literature, we now recommend the introduction of educational, clinical, management, and research activities designed to promote public awareness and cooperation, and to improve the safety and quality of care in the health system. The Ministry of Health, in collaboration with other government ministries, should initiate and integrate programs to improve patient engagement at all organizational levels. The activities in these areas should be focused on patient empowerment and literacy and should be implemented nationally at the level of policymakers, at an intermediate level of health organizations, and at an individual level comprising practitioners and healthcare consumers. Improving patient literacy regarding patient safety requires a combination of clear communication, education, engagement strategies, and system-wide support.

At the **level of policymakers**, the regulator should allocate a budget not only for the already on-going national programs for quality improvement but also for programs and campaigns tailored to the needs of the general public. At this organizational level, we propose the following system-wide initiatives:

- Establishment of patient advisory councils to include patient perspectives in safety programs.
- Development of educational and training programs for healthcare providers in health literacy best practices.
- Collecting data and conducting surveys to identify patient concerns and areas for improvement.
- Initiating and running public awareness campaigns addressing social media, posters, and newsletters to spread patient safety information.
- Establishment and celebration of Patient Safety Awareness Week addressed to the general public.
- Initiating partnerships with community organizations to extend reach beyond healthcare.

These campaigns should be conducted under the guidance and advice of experts in shaping public opinion and public relations. A public board of patients should advise the regulator about the focus of the programs and the ways to integrate the public as a partner in promoting safety of care.

We can also recommend policymakers and healthcare system leaders to implement precise instructions, guidelines, and procedures that ensure transparency in reporting and dealing with medical errors and explaining potential side effects of prescribed medications or procedures as well as patient identification. Specifically, a special program designed to bridge the deep ignorance gap of the public regarding the PRA (Patient Rights ACT) demonstrated in this study, should be developed and widely implemented. We believe that familiarity with their rights may impact the quality and safety of care of patients in Israel.

At the **intermediate level**, healthcare organization awareness programs should be specifically customized for the target audience: visitors; patients and their families; companions; and caregivers among others. The following steps can be implemented in health organizations:

- Implementation of checklists and reminders to reinforce safety behaviors.
- Development and implementation of patient education programs, workshops, and interactive learning sessions addressing major patient safety issues such as medication safety, infection prevention, and fall prevention.
- Creation of online modules and mobile apps for self-paced learning for the general public.
- Promotion and implementation of “Ask Me 3” (What is my main problem? What do I need to do? Why is it important?) campaigns.
- Promotion of patient portals offering easy access to test results, prescriptions, and patient safety tips.

At the **individual level**, tools designed to increase awareness of risks and emphasize the importance of patient involvement in their care must be developed and provided to workers at the patient’s bedside with the aim of preventing avoidable errors:

- Engaging patients in their care.
- Encouraging patients to ask questions and participate in shared decision-making.
- Implementing bedside communication and reporting so patients hear and confirm key details.
- Implementing teach-back methods, where patients repeat the information in their own words.
- Ensuring clear clinician-client communication by using plain language when explaining medical terms, risks, and procedures.
- Providing written materials at an appropriate reading level while utilizing visual aids (infographics, videos, diagrams) to enhance understanding.
- Offering culturally adapted services for non-native speakers.

The results of our study indicate that the current policies are insufficient and should provide more concrete proposals and activities. For instance, regarding the patient identification process, we must increase public awareness that this process should always be performed when receiving medications, specify who bears responsibility for patient education (e.g., public health campaigns, provider training), and establish how this education should be provided.

We can conclude that, in parallel with the clinical and administrative sectors, issues of patient safety, patient involvement, health literacy, shared decisions, and self-risk management should also be implemented among healthcare consumers and caregivers at various levels. Education of the general public should begin during mandatory schooling, and should then be continued in professional syllabi, by including a program for quality and safety training and assessment for healthcare professionals. This should include the standard provision of both verbal and written lists of possible complications and side effects when prescribing medications and before any invasive procedure.

## Conclusions

Patient safety and risk management are critical aspects of healthcare/medical care that have gained significant public attention in recent years. Patients and their families are now more knowledgeable about these issues and demand better standards of care. Healthcare providers are expected to adhere to high standards of care and incorporate patient safety and risk management education into their curricula. The healthcare industry has made significant strides in improving patient safety through organizations such as the National Patient Safety Foundation (NPSF), established in 1997, and the Institute for Healthcare Improvement (IHI), which has played a significant role in improving patient safety by developing programs and tools to reduce medical errors. Despite these efforts, patient safety remains a significant concern, and healthcare providers must improve their attitude and remain vigilant in identifying and minimizing risks associated with medical care. We suggest that the questionnaire we developed for this study should be adopted as a standard tool for exploring wide public attitudes towards patient safety and risk management. The findings will then serve as an important source of information for improvements in specific issues of patient safety plans, such as patient involvement, participation, and comprehension. We also recommend the inclusion of healthcare provider training in patient participation and education from school through continuing medical education programs. This should then be monitored to evaluate effectiveness.

## Data Availability

The data supporting this study’s findings are available from the corresponding author [IK] upon reasonable request.

## Declarations

### Reporting guidelines

STROBE reporting guidelines were used.

## References

[1] Smith CM. Origin and uses of primum Non nocere–above all, do no harm! J Clin Pharmacol. 2005;45:371–7. 15778417 10.1177/0091270004273680

[2] Moppett IK, Moppett SH. Surgical caseload and the risk of surgical never events in England. Anaesthesia. 2016;71:17–30. 26597516 10.1111/anae.13290

[3] Lee HJ, Jang SG, Choi JE, et al. Assessment of public perception regarding patient engagement for patient safety in Korea. J Patient Saf. 2021;17:44–50. 30633064 10.1097/PTS.0000000000000565 PMC7781086

[4] Madden C, Lydon S, Murphy AW, et al. Patients’ perception of safety climate in Irish general practice: a cross-sectional study. BMC Fam Pract. 2021;22:257. 34961484 10.1186/s12875-021-01603-9 PMC8710927

[5] Lasser EC, Heughan JA, Lai AY, et al. Patient perceptions of safety in primary care: a qualitative study to inform care. Curr Med Res Opin. 2021;37:1991–99. 34490810 10.1080/03007995.2021.1976736 PMC9721527

[6] Ricci-Cabello I, Pons-Vigués M, Berenguera A, et al. Patients’ perceptions and experiences of patient safety in primary care in England. Fam Pract. 2016;33:535–42. 27312563 10.1093/fampra/cmw046

[7] Villar V, Duarte S, Martins M. Patient safety in hospital care: a review of the patient’s perspective. Cadernos De Saude Publica. 2020;36:e00223019. 33331556 10.1590/0102-311X00223019

[8] Alhewiti A. Public perception of medical errors and confusion about medical complications: implications for healthcare safety in Saudi Arabia. Int J Gen Med. 2025;18:2093–106. 40236386 10.2147/IJGM.S517843 PMC11998984

[9] Hilleary RS, Jabusch SM, Zheng B, et al. Gender disparities in patient education provided during patient visits with a diagnosis of coronary heart disease. Women’s Health (London England). 2019;15:1745506519845591. 31106698 10.1177/1745506519845591 PMC6535750

[10] Wang Y, Hunt K, Nazareth I, et al. Do men consult less than women? An analysis of routinely collected UK general practice data. BMJ Open. 2013;3:e003320. 23959757 10.1136/bmjopen-2013-003320 PMC3753483

[11] Ledford CJ, Villagran MM, Kreps GL, et al. Practicing medicine: patient perceptions of physician communication and the process of prescription. Patient Educ Couns. 2010;80:384–92. 20675096 10.1016/j.pec.2010.06.033

[12] Glaser J, Nouri S, Fernandez A, et al. Interventions to improve patient comprehension in informed consent for medical and surgical procedures: an updated systematic review. Med Decis Making: Int J Soc Med Decis Mak. 2020;40:119–43. 10.1177/0272989X19896348 PMC7079202 31948345

[13] Taibanguay N, Chaiamnuay S, Asavatanabodee P, et al. Effect of patient education on medication adherence of patients with rheumatoid arthritis: a randomized controlled trial. Patient Prefer Adherence. 2019;13:119–29. 30666095 10.2147/PPA.S192008 PMC6333161

[14] Likic R, Maxwell SR. Prevention of medication errors: teaching and training. Br J Clin Pharmacol. 2009;67:656–61. 19594534 10.1111/j.1365-2125.2009.03423.x PMC2723205

[15] Institute for Healthcare Improvement. https://www.ihi.org/ accessed 1st January 2025.

[16] Mendlovic S, Roe D, Markusfeld G, et al. Exploring the relation between clinician ratings and patient-reported experience and outcomes. Int J Qual Health Care: J Int Soc Qual Health Care. 2022;34:ii98–104. 10.1093/intqhc/mzac004 35357441

[17] Physician-Patient Alliance for Health & Safety (PPAHS). Learning Improves Patient Safety and the Quality of Patient Care.https://ppahs.org/learning-center/?gclid=Cj0KCQjw2v-gBhC1ARIsAOQdKY3aXLWGP0Zadpnma1sN4V1aOSHBMJupSz7x0o6i-LsOcPdRNTsC7yMaAlViEALw_wcB accessed April 28, 2023.

[18] Ahmed FA, Asif F, Mubashir A, et al. Incorporating patient safety and quality into the medical school curriculum: an assessment of student gains. J Patient Saf. 2022;18:637–44. 35532980 10.1097/PTS.0000000000001010 PMC9422755

